# P05-11 Motor Competence Assessment of Czech School-Age Children: Lack of Movement or Developmental Coordination Disorder?

**DOI:** 10.1093/eurpub/ckac095.078

**Published:** 2022-08-29

**Authors:** Iva Šeflová

**Affiliations:** Faculty of Science, Humanities and Education, Technical University of Liberec, Liberec, Czech Republic

**Keywords:** Motor competence, developmental coordination disorder, Bruininks-Oseretsky Test

## Abstract

**Background:**

Physical activity (PA) in Czech children is insufficient. In the last two decades, the prevalence of physical inactivity and excessive time spent in sedentary activities has increased (Gába et al. 2019). The determinants of PA are complex and wide-ranging: individual, socio-demographic, interpersonal, environmental. Motor skill acquisition in early childhood may be an important prerequisite for child PA participation and engagement in PA later in life (Loprinzi et al. 2012).

The aim of this study was to estimate motor competence (MC) level in a Czech school children, identify children with motor impairments and analyze the possible causes.

**Methods:**

The research sample was made from Czech school children (n = 195, 110 girls, and 85 boys) of average age 11.96±1.96 years. To estimate an MC, we used the Bruininks-Oseretsky Test of Motor Proficiency, Second Edition, a complete form. We evaluated total motor composite (TMC) and four subcategories: fine manual control, manual coordination, body coordination, and strength and agility.

**Results:**

Our TMC results correspond to a secular reduction in MC. The results show that the group's MC is in the lower part of the average level (TMC standard score 45.4±11.7). The overall percentage of children whose TMC is above the 15th percentile is 64.6%. Conversely, 35.4% of children do not meet the criterion in TMC.

On average, the weakest performance was recorded in the area of fine manual control (standard score 38.2±11.6). More in-depth analysis showed that the weakest subcomponent of fine manual control was fine motor precision (scale score 9.3±5.8) and fine motor integration (scale score 10.7±6.1).

**Conclusion:**

24 children (12.3%) had well-below average results (TMC<5th percentile). These children are highly likely to develop a developmental coordination disorder. It is a significantly higher result than in the literature reported around 5 - 6% in school-age children.

69 children (35.4%) had below-average result of TMC (TMC<15th percentile). The poor results in the fine manual control without the accompanying other motor components point to a low exercise experience rather than a neurodevelopmental disorder.

